# Supplementary material for: Complex hydrothermal vent microbial mat communities used to assess primer selection for targeted amplicon surveys from Kama‘ehuakanaloa Seamount
Source: PeerJ. 2024 Sep 16;12:e18099. doi: 10.7717/peerj.18099 (PMC11412224; doi:10.7717/peerj.18099)
Supplement: Supplemental Information 3 [file peerj-12-18099-s003.docx]

| ***r*_pb_** | ***p*-value** | **Domain** | **Phylum** | **Class** | **V3V4 Reads** |
| --- | --- | --- | --- | --- | --- |
| 0.845 | 0.0004 | Bacteria | Acetothermia | Acetothermiia | 5221 |
| 0.777 | 0.0004 | Bacteria | Patescibacteria | Microgenomatia | 2198 |
| 0.735 | 0.0004 | Bacteria | Proteobacteria | Gammaproteobacteria | 174 |
| 0.721 | 0.0004 | Bacteria | Planctomycetota | Planctomycetes | 7097 |
| 0.705 | 0.0004 | Bacteria | Actinobacteriota | Acidimicrobiia | 403 |
| 0.698 | 0.0004 | Bacteria | Patescibacteria | ABY1 | 1931 |
| 0.667 | 0.0004 | Bacteria | Patescibacteria | Parcubacteria | 1515 |
| 0.665 | 0.0013 | Bacteria | Proteobacteria | Alphaproteobacteria | 89 |
| 0.647 | 0.0013 | Bacteria | Bacteroidota | Unclassified | 4220 |
| 0.644 | 0.0259 | Bacteria | Myxococcota | Polyangia | 181 |
| 0.642 | 0.0110 | Bacteria | Proteobacteria | Gammaproteobacteria | 132 |
| 0.620 | 0.0058 | Bacteria | Verrucomicrobiota | Chlamydiae | 34 |
| 0.619 | 0.0012 | Bacteria | Patescibacteria | ABY1 | 15791 |
| 0.617 | 0.0064 | Bacteria | Proteobacteria | Alphaproteobacteria | 915 |
| 0.617 | 0.0209 | Bacteria | Acidobacteriota | Unclassified | 138 |
| 0.616 | 0.0144 | Bacteria | Acidobacteriota | Acidobacteriae | 209 |
| 0.597 | 0.0006 | Bacteria | Patescibacteria | Parcubacteria | 944 |
| 0.575 | 0.0086 | Bacteria | Verrucomicrobiota | Verrucomicrobiae | 132 |
| 0.557 | 0.0231 | Bacteria | Patescibacteria | ABY1 | 1312 |
| 0.547 | 0.0213 | Bacteria | Patescibacteria | Gracilibacteria | 1882 |
| 0.546 | 0.0324 | Bacteria | Calditrichota | Calditrichia | 4349 |
| 0.533 | 0.0355 | Bacteria | Patescibacteria | Parcubacteria | 213 |
| 0.524 | 0.0480 | Bacteria | Patescibacteria | Gracilibacteria | 180 |
| 0.520 | 0.0013 | Bacteria | Patescibacteria | ABY1 | 729 |
| 0.516 | 0.0017 | Bacteria | Proteobacteria | Gammaproteobacteria | 110 |
| 0.514 | 0.0008 | Bacteria | Patescibacteria | Gracilibacteria | 53836 |
| 0.514 | 0.0246 | Bacteria | Proteobacteria | Gammaproteobacteria | 541 |
| 0.512 | 0.0004 | Bacteria | Planctomycetota | Planctomycetes | 8660 |
| 0.512 | 0.0004 | Bacteria | Patescibacteria | Parcubacteria | 269 |
| 0.510 | 0.0170 | Bacteria | Patescibacteria | ABY1 | 4854 |
| 0.494 | 0.0159 | Bacteria | Proteobacteria | Alphaproteobacteria | 313 |
| 0.489 | 0.0004 | Bacteria | Desulfobacterota | Desulfuromonadia | 4657 |
| 0.486 | 0.0073 | Bacteria | Patescibacteria | ABY1 | 751 |
| 0.486 | 0.0004 | Bacteria | Patescibacteria | Parcubacteria | 193 |
| 0.480 | 0.0244 | Bacteria | Campilobacterota | Campylobacteria | 1254 |
| 0.472 | 0.0262 | Bacteria | Desulfobacterota | Syntrophobacteria | 3707 |
| 0.470 | 0.0004 | Bacteria | Actinobacteriota | Acidimicrobiia | 1380 |
| 0.469 | 0.0006 | Bacteria | Verrucomicrobiota | Chlamydiae | 1887 |
| 0.469 | 0.0260 | Bacteria | Bacteroidota | Bacteroidia | 33 |
| 0.463 | 0.0005 | Bacteria | Patescibacteria | ABY1 | 10276 |
| 0.463 | 0.0004 | Bacteria | Actinobacteriota | Acidimicrobiia | 355 |
| 0.454 | 0.0450 | Bacteria | Patescibacteria | Microgenomatia | 671 |
| 0.453 | 0.0004 | Bacteria | Bacteroidota | Bacteroidia | 429 |
| 0.441 | 0.0239 | Bacteria | Proteobacteria | Gammaproteobacteria | 90 |
| 0.423 | 0.0366 | Bacteria | Actinobacteriota | Acidimicrobiia | 123 |
| 0.375 | 0.0187 | Bacteria | Proteobacteria | Gammaproteobacteria | 1738 |
| 0.375 | 0.0259 | Bacteria | Patescibacteria | Parcubacteria | 290 |
| 0.334 | 0.0061 | Bacteria | Campilobacterota | Campylobacteria | 593 |
| 0.320 | 0.0064 | Bacteria | Proteobacteria | Gammaproteobacteria | 65 |
| 0.264 | 0.0004 | Bacteria | Proteobacteria | Gammaproteobacteria | 14793 |
