# Supplementary material for: Complex hydrothermal vent microbial mat communities used to assess primer selection for targeted amplicon surveys from Kama‘ehuakanaloa Seamount
Source: PeerJ. 2024 Sep 16;12:e18099. doi: 10.7717/peerj.18099 (PMC11412224; doi:10.7717/peerj.18099)
Supplement: Supplemental Information 4 [file peerj-12-18099-s004.docx]

| ***r*_pb_** | ***p*-value** | **Domain** | **Phylum** | **Class** | **V4V5 Reads** |
| --- | --- | --- | --- | --- | --- |
| 0.839 | 0.0004 | Bacteria | Planctomycetota | 028H05-P-BN-P5 | 65 |
| 0.815 | 0.0004 | Bacteria | Proteobacteria | Gammaproteobacteria | 178 |
| 0.759 | 0.0008 | Bacteria | Chloroflexi | Anaerolineae | 2096 |
| 0.756 | 0.0004 | Archaea | Crenarchaeota | Nitrososphaeria | 12947 |
| 0.743 | 0.0004 | Bacteria | Proteobacteria | Gammaproteobacteria | 638 |
| 0.726 | 0.0004 | Archaea | Crenarchaeota | Nitrososphaeria | 5372 |
| 0.708 | 0.0037 | Bacteria | PAUC34f | Unclassified | 511 |
| 0.696 | 0.0004 | Bacteria | Proteobacteria | Gammaproteobacteria | 441 |
| 0.691 | 0.0006 | Bacteria | Proteobacteria | Gammaproteobacteria | 1050 |
| 0.683 | 0.0004 | Bacteria | Planctomycetota | Phycisphaerae | 4590 |
| 0.683 | 0.0058 | Bacteria | Proteobacteria | Unclassified | 8055 |
| 0.677 | 0.0004 | Bacteria | Proteobacteria | Alphaproteobacteria | 125 |
| 0.668 | 0.0037 | Bacteria | Bacteroidota | Bacteroidia | 196 |
| 0.650 | 0.0004 | Bacteria | Acidobacteriota | Vicinamibacteria | 153 |
| 0.643 | 0.0004 | Bacteria | Proteobacteria | Gammaproteobacteria | 174 |
| 0.640 | 0.0099 | Bacteria | Desulfobacterota | Desulfuromonadia | 7181 |
| 0.636 | 0.0011 | Bacteria | Gemmatimonadota | BD2-11 | 658 |
| 0.633 | 0.0070 | Bacteria | Proteobacteria | Gammaproteobacteria | 153 |
| 0.630 | 0.0010 | Bacteria | Bacteroidota | Kryptonia | 9136 |
| 0.622 | 0.0129 | Bacteria | Gemmatimonadota | Unclassified | 57 |
| 0.615 | 0.0066 | Bacteria | Proteobacteria | Gammaproteobacteria | 78 |
| 0.613 | 0.0014 | Bacteria | AncK6 | Unclassified | 409 |
| 0.591 | 0.0014 | Bacteria | Verrucomicrobiota | Lentisphaeria | 86 |
| 0.590 | 0.0004 | Bacteria | Verrucomicrobiota | Omnitrophia | 173 |
| 0.584 | 0.0005 | Archaea | Nanoarchaeota | Nanoarchaeia | 2566 |
| 0.575 | 0.0121 | Bacteria | Bacteroidota | Ignavibacteria | 38647 |
| 0.571 | 0.0004 | Bacteria | Hydrogenedentes | Hydrogenedentia | 175 |
| 0.569 | 0.0010 | Bacteria | Proteobacteria | Gammaproteobacteria | 774 |
| 0.566 | 0.0004 | Bacteria | Proteobacteria | Alphaproteobacteria | 14900 |
| 0.564 | 0.0211 | Bacteria | Bacteroidota | Bacteroidia | 583 |
| 0.563 | 0.0022 | Bacteria | Verrucomicrobiota | Lentisphaeria | 361 |
| 0.561 | 0.0004 | Bacteria | Proteobacteria | Alphaproteobacteria | 1053 |
| 0.553 | 0.0256 | Bacteria | Desulfobacterota | Desulfuromonadia | 47 |
| 0.553 | 0.0260 | Bacteria | Proteobacteria | Alphaproteobacteria | 120 |
| 0.550 | 0.0229 | Bacteria | Bacteroidota | Bacteroidia | 366 |
| 0.547 | 0.0264 | Bacteria | Proteobacteria | Zetaproteobacteria | 990865 |
| 0.540 | 0.0326 | Bacteria | Nitrospirota | BMS9AB35 | 28284 |
| 0.538 | 0.0329 | Bacteria | Planctomycetota | BD7-11 | 3262 |
| 0.536 | 0.0238 | Bacteria | Proteobacteria | Gammaproteobacteria | 19 |
| 0.532 | 0.0235 | Bacteria | Bacteroidota | Bacteroidia | 25 |
| 0.530 | 0.0309 | Bacteria | Bdellovibrionota | Bdellovibrionia | 3130 |
| 0.526 | 0.0407 | Bacteria | Nitrospinota | Nitrospinia | 7061 |
| 0.522 | 0.0259 | Bacteria | Myxococcota | Myxococcia | 94 |
| 0.522 | 0.0067 | Bacteria | Proteobacteria | Gammaproteobacteria | 116 |
| 0.520 | 0.0010 | Archaea | Asgardarchaeota | Heimdallarchaeia | 166 |
| 0.519 | 0.0273 | Archaea | Asgardarchaeota | Lokiarchaeia | 90 |
| 0.515 | 0.0433 | Bacteria | Chloroflexi | Chloroflexia | 476 |
| 0.515 | 0.0413 | Bacteria | Unclassified | Unclassified | 102035 |
| 0.509 | 0.0010 | Bacteria | Chloroflexi | Chloroflexia | 1049 |
| 0.500 | 0.0007 | Bacteria | Deinococcota | Deinococci | 3676 |
| 0.495 | 0.0019 | Bacteria | Verrucomicrobiota | Verrucomicrobiae | 247 |
| 0.490 | 0.0045 | Bacteria | Desulfobacterota | Unclassified | 11101 |
| 0.486 | 0.0016 | Bacteria | Proteobacteria | Alphaproteobacteria | 19849 |
| 0.481 | 0.0060 | Bacteria | Proteobacteria | Alphaproteobacteria | 547 |
| 0.479 | 0.0060 | Bacteria | Bacteroidota | Bacteroidia | 381 |
| 0.478 | 0.0004 | Bacteria | Proteobacteria | Gammaproteobacteria | 1245 |
| 0.476 | 0.0273 | Bacteria | Chloroflexi | Anaerolineae | 126 |
| 0.471 | 0.0023 | Bacteria | Bacteroidota | Ignavibacteria | 3042 |
| 0.464 | 0.0251 | Bacteria | Acidobacteriota | Thermoanaerobaculia | 738 |
| 0.462 | 0.0010 | Bacteria | Desulfobacterota | Desulfarculia | 172 |
| 0.462 | 0.0239 | Bacteria | Spirochaetota | Leptospirae | 1861 |
| 0.460 | 0.0015 | Bacteria | Bacteroidota | Bacteroidia | 148 |
| 0.454 | 0.0273 | Bacteria | Chloroflexi | Dehalococcoidia | 6015 |
| 0.454 | 0.0372 | Bacteria | Marinimicrobia | Unclassified | 130 |
| 0.451 | 0.0206 | Bacteria | Planctomycetota | Planctomycetes | 183 |
| 0.449 | 0.0060 | Bacteria | Spirochaetota | Spirochaetia | 1587 |
| 0.433 | 0.0453 | Bacteria | Proteobacteria | Gammaproteobacteria | 79 |
| 0.424 | 0.0230 | Bacteria | Actinobacteriota | Coriobacteriia | 209 |
| 0.409 | 0.0045 | Bacteria | Dependentiae | Babeliae | 189 |
| 0.390 | 0.0004 | Bacteria | Bacteroidota | Bacteroidia | 11087 |
| 0.382 | 0.0134 | Bacteria | Planctomycetota | Planctomycetes | 437 |
| 0.325 | 0.0461 | Archaea | Halobacterota | Unclassified | 1570 |
| 0.269 | 0.0004 | Bacteria | Proteobacteria | Gammaproteobacteria | 7319 |
