# Supplementary figures and images for: Complex hydrothermal vent microbial mat communities used to assess primer selection for targeted amplicon surveys from Kama‘ehuakanaloa Seamount
Source: PeerJ. 2024 Sep 16;12:e18099. doi: 10.7717/peerj.18099 (PMC11412224; doi:10.7717/peerj.18099)

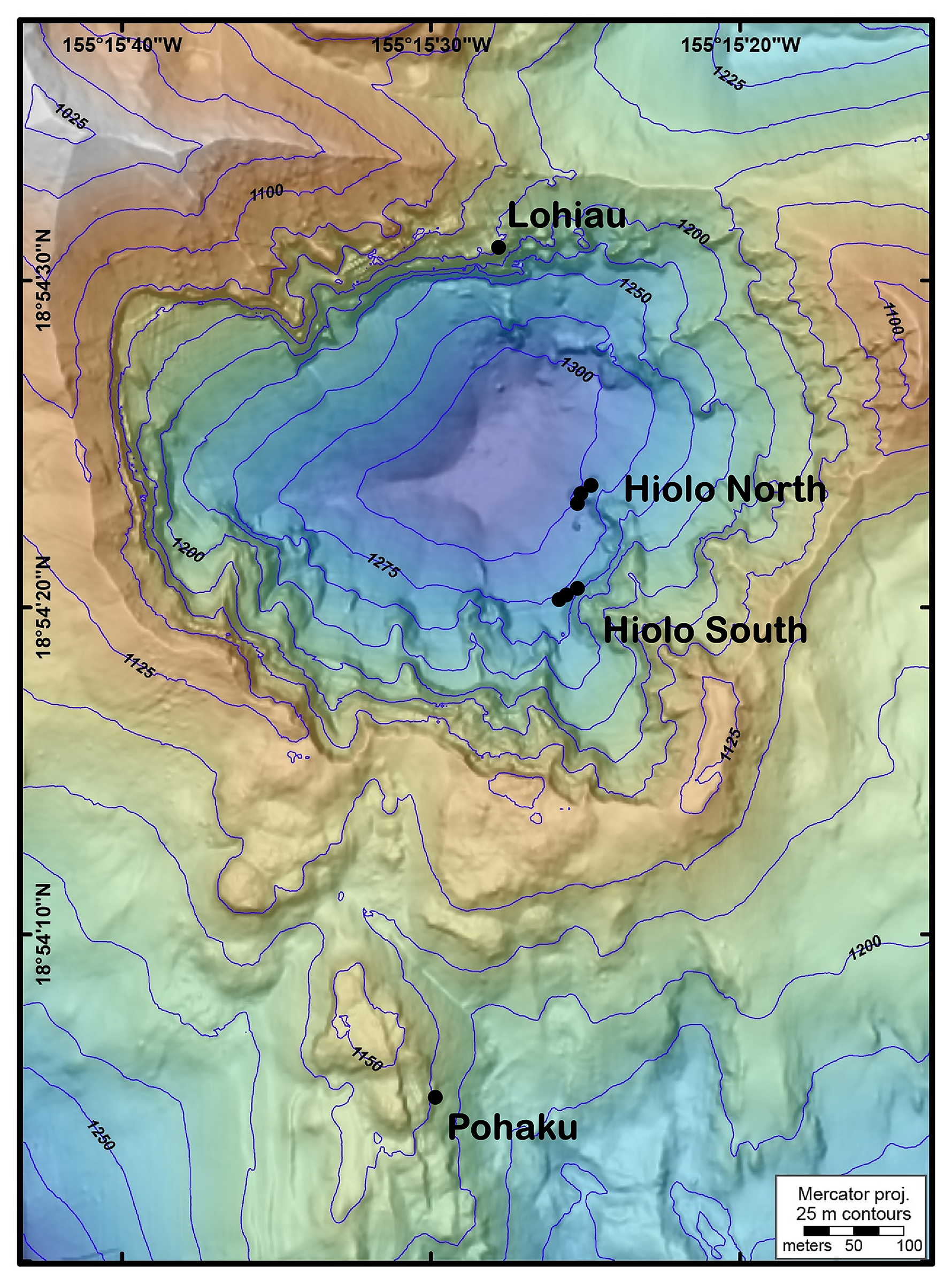

Supplement: Supplemental Information 5 — Bathymetric map (high resolution at <2 m) of sampling sites in and near Pele’s Pit caldera on the summit of Kama‘ehuakanaloa Seamount, Hawai’i. Precise marker locations include Pohaku (Marker 57), Lohiau (Marker 2), Hiolo North (Markers 36, 39 and 31), and Hiolo South (Markers 34, 38 and Ku’kulu). Courtesy of Susan Merle, NOAA EOI/OSU (Clague et al., 2019). [file peerj-12-18099-s005.png]

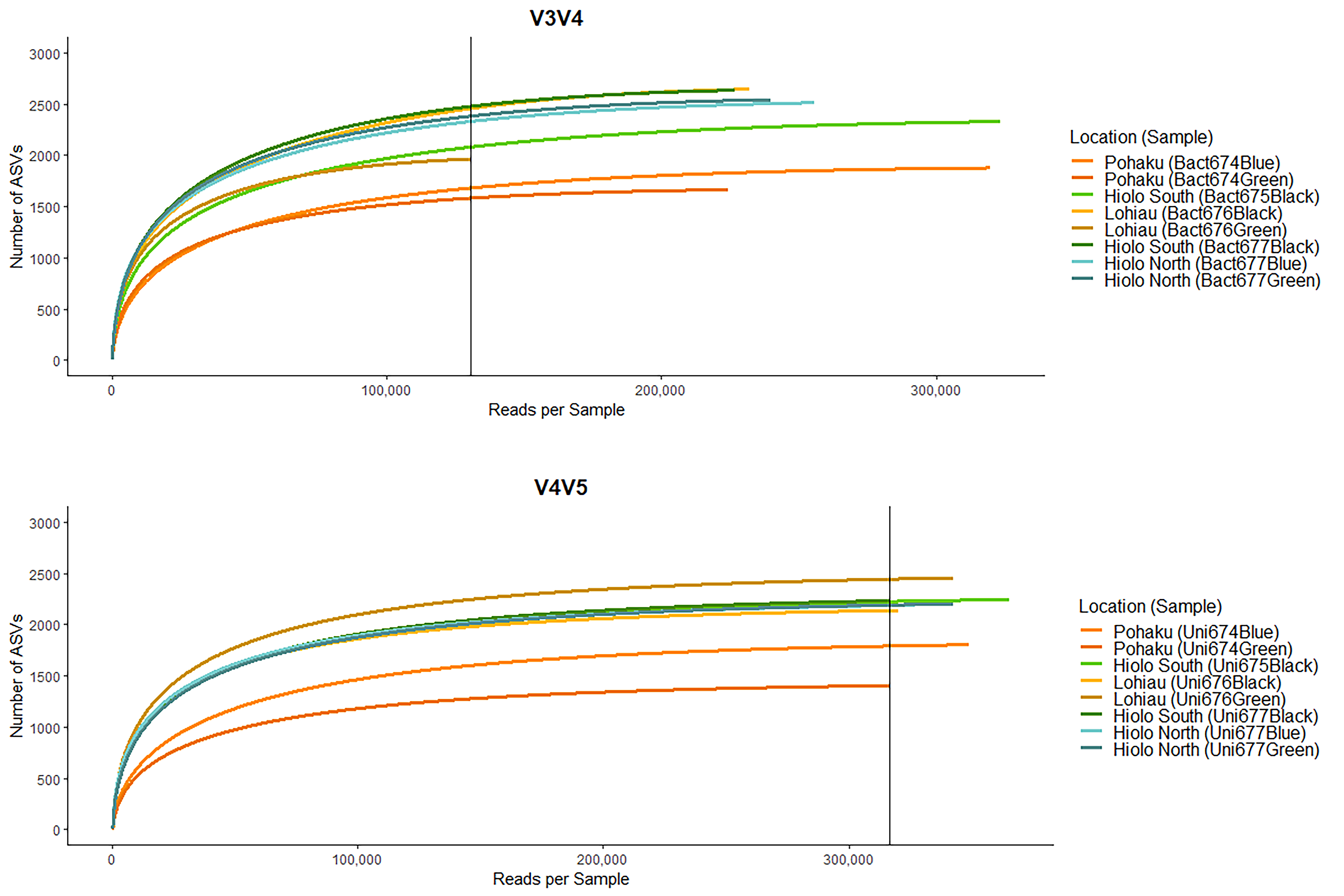

Supplement: Supplemental Information 6 — Rarefaction curves for each microbial mat community, colored by sample location for both primer sets. Vertical lines denote the minimum read depth. NB: The difference in the x-axis scales. [file peerj-12-18099-s006.png]
